# Highly variable mRNA half‐life time within marine bacterial taxa and functional genes

**DOI:** 10.1111/1462-2920.14737

**Published:** 2019-07-25

**Authors:** Paul A. Steiner, Daniele De Corte, Javier Geijo, Catalina Mena, Taichi Yokokawa, Thomas Rattei, Gerhard J. Herndl, Eva Sintes

**Affiliations:** ^1^ Limnology and Bio‐Oceanography University of Vienna, Althanstrasse 14, 1090 Vienna Austria; ^2^ Marine Functional Biology Group Research and Development Center for Marine Biosciences, Japan Agency for Marine‐Earth Science and Technology (JAMSTEC), Natushima 2‐15 Yokosuka Kanagawa Japan; ^3^ Department of Microbiology and Ecosystem Science, Division of Computational Systems Biology University of Vienna, Althanstrasse 14, 1090 Vienna Austria; ^4^ Instituto Español de Oceanografia Centre Oceanogràfic de les Balears, Moll de Ponent s/n, 07015 Palma Spain; ^5^ Institute for Extra‐cutting‐edge Science and Technology Avant‐garde Research (X‐star) Japan Agency for Marine‐Earth Science and Technology (JAMSTEC), Natushima 2‐15 Yokosuka Kanagawa Japan; ^6^ Department of Marine Microbiology and Biogeochemistry Royal Netherlands Institute for Sea Research, Utrecht University PO Box 59, Alberta Den Burg, 1790 The Netherlands

## Abstract

Messenger RNA can provide valuable insights into the variability of metabolic processes of microorganisms. However, due to uncertainties that include the stability of RNA, its application for activity profiling of environmental samples is questionable. We explored different factors affecting the decay rate of transcripts of three marine bacterial isolates using qPCR and determined mRNA half‐life time of specific bacterial taxa and of functional genes by metatranscriptomics of a coastal environmental prokaryotic community. The half‐life time of transcripts from 11 genes from bacterial isolates ranged from 1 to 46 min. About 80% of the analysed transcripts exhibited half‐live times shorter than 10 min. Significant differences were found in the half‐life time between mRNA and rRNA. The half‐life time of mRNA obtained from a coastal metatranscriptome ranged from 9 to 400 min. The shortest half‐life times of the metatranscriptome corresponded to transcripts from the same clusters of orthologous groups (COGs) in all bacterial classes. The prevalence of short mRNA half‐life time in genes related to defence mechanisms and motility indicate a tight connection of RNA decay rate to environmental stressors. The short half‐life time of RNA and its high variability needs to be considered when assessing metatranscriptomes especially in environmental samples.

## Introduction

In contrast to the genome, which illustrates the metabolic potential of microorganisms, the transcriptome indicates metabolic activity. Identification and quantification of expressed genes at a particular moment can provide valuable insights into the response of organisms to environmental conditions (Cottrell and Kirchman, 2016) or microbial community dynamics (McCarren *et al*., 2010). However, poor correlations between mRNA and protein levels raised concerns about the use of mRNA for activity profiling (Greenbaum *et al*., 2003; Wang *et al*., 2019). Moreover, capturing the expression profile of an organism at a specific moment is typically challenging due to the rapid turnover and decay of mRNA (Hambraeus *et al*., 2003; Selinger *et al*., 2003; Steglich *et al*., 2010; Kristoffersen *et al*., 2012). RNA half‐life times (T_1/2_) as short as a few seconds for single transcripts (Steglich *et al*., 2010) and few minutes for cultured prokaryotic species (Hambraeus *et al*., 2003; Selinger *et al*., 2003; Steglich *et al*., 2010) have been reported. However, stable mRNAs have also been observed (Hambraeus *et al*., 2003), indicating a high complexity of the mechanisms regulating RNA degradability. RNA T_1/2_ has been mostly determined on model microorganisms such as *Escherichia coli* and *Bacillus subtilis* to understand the mechanisms causing the instability (Jain, 2002; Kushner, 2002; Condon, 2003). Ribonucleases (RNAses) are main players in RNA metabolism and enable controlled degradation of RNA (Deutscher, 2006, 2015). One of the cells' strategies to deal with RNAses is to isolate them from the RNA molecules by locating RNAses in the periplasmic space, bound to the inner cell membrane or in the cell periphery (Taghbalout *et al*., 2014; Deutscher, 2015). However, after cell lysis or damage, RNAses can get access and degrade RNA molecules in an uncontrolled way, both inside and outside the cell (Deutscher, 2015). A multiprotein complex termed RNA degradosome has been shown to play a major role in RNA degradation (Deutscher, 2006). Other factors such as RNA type (Deutscher, 2003, 2006) and gene position (Hambraeus *et al*., 2003; Steglich *et al*., 2010) suggest a structural base in the RNA degradation patterns. However, less attention was given to environmental factors, such as temperature, and whether or how it influences RNA decay (as reviewed in (Takayama and Kjelleberg, 2000). Reports of both extremely short decay rates and stable mRNAs in selected organisms, for example, the longer half‐life times reported for archaeal mRNAs as compared to bacterial mRNAs (Clouet‐d'Orval *et al*., 2018) indicate potentially high variability of RNA decay rates within complex communities.

In this study, we analysed RNA degradation patterns of three marine bacterial isolates and related RNA T_1/2_ to RNA type (mRNA, rRNA), optimal growth temperature and growth rate. Furthermore, we used metatranscriptomics to assess the mRNA T_1/2_ from a coastal prokaryotic community on a phylogenetic and a functional level to examine possible ecological factors such as competition or nutrient stress causing RNA degradation and determining RNA degradation rates.

## Results and discussion

### *RNA half‐life time of bacterial isolates determined by qPCR*

The three bacterial isolates had a query cover of 99% to 100% and an identity between 98% and 99% to *Alcanivorax jadensis* (Accession Nr.: NR_025271.1), *Colwellia polaris* (Accession Nr.: NR_043462.1) and *Croceibacter atlanticus* (Accession Nr.: NR_074636.1). The respective isolates are subsequently referred to as *Alcanivorax*, *Colwellia* and *Croceibacter. Alcanivorax* and *Croceibacter* are marine bacteria typically found in the water column of the Mediterranean Sea and Atlantic (Cho and Giovannoni, 2003; Fernández‐Martínez *et al*., 2003). *Colwellia polaris* is a psychrotolerant marine bacterium first isolated from Arctic sea‐ice (Cho and Giovannoni, 2003; Fernández‐Martínez *et al*., 2003; Zhang *et al*., 2008).

RNA degradation was assessed during the early stationary phase of the isolates grown at 4, 15, and 25 °C. At the early stationary phase, cell abundance varied between 3.7 × 10^8^ and 8.0 × 10^8^ cells mL^−1^ in *Alcanivorax* cultures, 3.8 × 10^7^ and 6.6 × 10^8^ cells mL^−1^ in *Colwellia*, and 1.5 × 10^9^ and 1.8 × 10^9^ cells mL^−1^ in *Croceibacter* cultures (Supporting Information Fig. S1).

**Figure 1:**
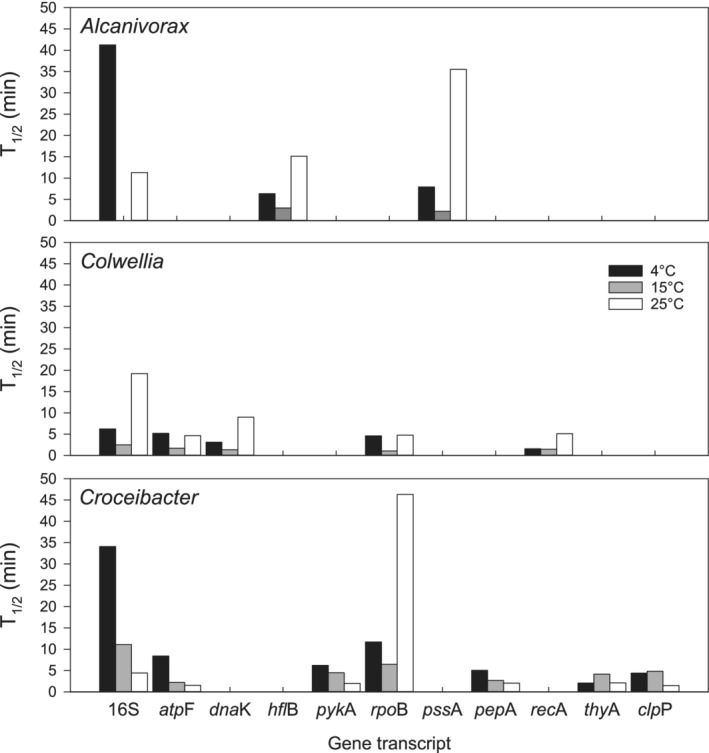
Half‐life time (T_1/2_) of gene transcripts of the genes 16SrRNA, *atp*F (ATP synthase B chain), *dna*K (chaperone Hsp70), *hfl*B (ATP‐dependent protease), *pyk*A (pyruvate kinase), *rpo*B (RNA polymerase ß subunit), *pss*A (phosphatidylserine synthase), *pep*A (aminopeptidase A/I), *rec*A (DNA repair protein), *thy*A (thymidylate synthase) and *clp*P (clp protease subunit 2) of the bacterial isolates *Alcanivorax jadensis* (*n* = 9), *Colwellia polaris* (*n* = 15) and *Croceibacter atlanticus* (*n* = 21) at 4, 15 and 25°C.

Overall, the T_1/2_ of transcripts of 11 genes varied from 1 min up to 46 min at all temperatures, as revealed by qPCR (Fig. 1 and Supporting Information Fig. S2). Eighty percent had a T_1/2_ shorter than 10 min, in agreement with the short half‐life times previously reported for bacterial RNA (Hambraeus *et al*., 2003; Selinger *et al*., 2003; Steglich *et al*., 2010; Kristoffersen *et al*., 2012).

The T_1/2_ of 16S rRNA was significantly longer than of the mRNAs at all temperatures, except for *Alcanivorax* at 25°C (Table 1). A higher stability of non‐coding RNA (ncRNA) as compared to mRNA has also been found in previous studies (Deutscher, 2003, 2006; Steglich *et al*., 2010). The mechanisms of resistance of ncRNA to degradation are unclear but likely due to physical protection (Deutscher, 2003, 2006). Different degradation patterns were observed at different temperatures. At 4 and 15°C, the T_1/2_ of *hfl*B and *pss*A of *Alcanivorax* was shorter (2.2 to 7.9 min) than of 16S rRNA T_1/2_ (>40 min), while at 25°C, a longer T_1/2_ for these two genes was obtained than for 16S rRNA T_1/2_ (Fig. 1 and Table 1). Such contrasting T_1/2_ patterns of mRNA and rRNA at different temperatures might arise from the flexibility and adaptability of the degradosome to environmental conditions (Prud'homme‐Généreux *et al*., 2004; Deutscher, 2006). All components of the degradosome need to function to ensure normal mRNA turnover (Bernstein *et al*., 2004). Different transcript types have been shown to be affected differently by failures in degradosome components (Bernstein *et al*., 2004). The long T_1/2_ of mRNA in *Alcanivorax* at 25 °C might result from temperature sensitivity of some components of the degradosome machinery (Sulthana *et al*., 2016). Typically 16S rRNA is degraded only under stress or in response to misassembly and deficiency (Deutscher, 2003; Sulthana *et al*., 2016). The degradation of 16S rRNA determined in this experiment might have been caused by the addition of the antibiotic rifampicin or, alternatively, temperature stress to the bacterial isolates (Sulthana *et al*., 2016).

**Table 1:** Half‐life time (T_1/2_) of transcripts of specific genes for the different isolates (Alc. = *Alcanivorax*, Col. = *Colwellia*, Cro. = *Croceibacter*) at 4, 15 and 25°C. Slope (m) coefficient of determination (*R* ^2^) of the fitted curve is also indicated. The *p*‐value (one‐sample *t*‐test) denoting the significance of the difference between the half‐life time of the 16S rRNA and other genes is also shown. Asterisk (*) indicates that 100 min was replaced by ‘no decay’ in the statistical test. Two asterisks (**) indicate that the T_1/2_ of *rpo*B was excluded from the one‐sample *t*‐test.

Gene		*Alc*. 4°C	A*lc*. 15°C	*Alc*. 25°C	*Col*. 4°C	*Col*. 15°C	*Col*. 25°C	*Cro*. 4°C	*Cro*. 15°C	*Cro*. 25°C
	*R* ^2^	0.32	0.10	0.58	0.59	0.22	0.92	0.70	1.00	0.88
16S rRNA	m	−0.01	0.01	−0.05	−0.10	−0.21	−0.03	−0.02	−0.06	−0.17
	T_1/2_	41.25	no decay	11.29	6.23	2.46	19.22	34.09	11.06	4.41
	*R* ^2^				0.91	0.52	0.94	0.99	0.99	0.99
*atp*F	m				−0.14	−0.35	−0.15	−0.08	−0.32	−0.45
	T_1/2_				5.17	1.69	4.63	8.39	2.20	1.51
	*R* ^2^				0.60	0.50	0.997			
*dna*K	m				−0.20	−0.44	−0.13			
	T_1/2_				3.07	1.32	8.97			
	*R* ^2^	0.99	0.99	0.98						
*hfl*B	m	−0.22	−0.23	−0.05						
	T_1/2_	6.33	2.98	15.15						
	*R* ^2^							0.97	0.90	0.99
*pyk*A	m							−0.12	−0.15	−0.36
	T_1/2_							6.20	4.47	1.97
	*R* ^2^				1.00	0.90	0.99	1.00	0.94	0.98
*rpo*B	m				−0.15	−0.72	−0.15	−0.06	−0.10	−0.02
	T_1/2_				4.57	1.03	4.73	11.70	6.45	46.34
	*R* ^2^	0.99	0.92	0.98						
*pss*A	m	−0.09	−0.34	−0.02						
	T_1/2_	7.91	2.19	35.51						
	*R* ^2^							0.86	0.72	0.96
*pep*A	m							−0.14	−0.23	−0.36
	T_1/2_							5.04	2.65	2.01
	*R* ^2^				0.94	0.36	0.93			
*rec*A	m				−0.47	−0.38	−0.13			
	T_1/2_				1.54	1.45	5.10			
	*R* ^2^							0.98	0.90	0.95
*thy*A	m							−0.36	−0.18	−0.32
	T_1/2_							2.06	4.16	2.11
	*R* ^2^							0.99	0.76	0.96
*clp*P	m							−0.16	−0.13	−0.49
	T_1/2_							4.37	4.81	1.47
*t*‐test	*p* value	0.01	0.003*	0.40	0.05	0.004	0.001	0.000	0.000	0.00004**

It has been hypothesized that the rate of transcription and hence, the growth rate of an organism, affects the T_1/2_ of mRNA (Vytvytska *et al*., 1998; Takayama and Kjelleberg, 2000). However, it has also been suggested that the T_1/2_ depends more on temperature than on growth rate (Hundt *et al*., 2007). The generation time of *Alcanivorax* correlated inversely to temperature (*r* = −0.97, *p* = 0.001, *n* = 6). The shortest generation time (3.17 h) was obtained at 30 °C and the longest generation time (13.32 h) at 4 °C (Supporting Information Fig. S3), in agreement with the classification of *Alcanivorax jadensis* as mesophilic in ‘The Bacterial Diversity Metadatabase’ (Söhngen *et al*., 2015) of the Leibniz Institute DMSZ. Consequently, the average mRNA T_1/2_ of *Alcanivorax* was long when generation time was short (*r* = −0.48, *p* = 0.68, *n* = 3) and temperature was high (*r* = 0.74, *p* = 0.47, *n* = 3) and *vice versa*. This is in contrast to the findings reported in other studies (Vytvytska *et al*., 1998; Hundt *et al*., 2007).

*Colwellia*, however, exhibited the shortest generation time (3.77 h) at 15 °C and long generation times at 4 and 25 °C (11.68 h and 9.50 h, respectively) (Supporting Information Fig. S3). It has been shown previously that *Colwellia polaris* reached its growth optimum at 20‑21 °C (Zhang *et al*., 2008). The increase of generation time beyond 15 °C indicates a psychrophilic life style of *Colwellia polaris*. In *C. polaris*, this is also indicated by ceased growth at 26 °C in a previous study (Zhang *et al*., 2008) and at 30 °C in this study (Supporting Information Fig. S3). The average T_1/2_ of transcripts of four genes (*atp*F, *dna*K, *rpo*B and *rec*A) of *C. polaris* were more strongly correlated with generation time (*r* = 0.69, *p* = 0.51, *n* = 3) than with temperature (*r* = 0.48, *p* = 0.68, *n* = 3), as both average T_1/2_ and generation time were shortest at 15 °C (Supporting Information Fig. S3). The long T_1/2_ at high temperatures might result from a malfunctioning degradosome.

The generation times of *Croceibacter* correlated inversely to temperature (*r* = −0.80, *p* = 0.06, *n* = 6) with the longest generation time (14.05 h) obtained at 4°C (Supporting Information Fig. S3). However, generation times were similar at 15 and 30 °C averaging 4.48 ± 0.47 h (mean ± SD), indicating a wide range of optimal temperature growth. The type strain *Croceibacter atlanticus* has an optimum growth temperature of 20–23 °C (Krieg *et al*., 2010) and, in contrast to our isolate, does not grow at 4 or 30 °C. The average T_1/2_ of six gene transcripts (*atp*F, *pyk*A, *rpo*B, *pep*A, *thy*A and *clp*P) of *Croceibacter* correlated negatively to temperature (*r* = −0.999, *p* = 0.03, *n* = 3) and positively to generation time (*r* = 0.89, *p* = 0.30, *n* = 3).

Correlations of T_1/2_ of individual gene transcripts to temperature and generation time (Supporting Information Table S1) varied strongly among the bacterial strains indicating that mRNA T_1/2_ is probably regulated by a combined effect of temperature and generation time among other factors such as the position within an operon (Hambraeus *et al*., 2003; Steglich *et al*., 2010) or the secondary structure and interactions with other molecules (Hambraeus *et al*., 2003).

### *Variability of RNA T1/2 in complex environmental communities determined by transcriptomics*

The number of reads ranged between 3.2 × 10^5^ and 2.2 × 10^6^ per metatranscriptome and the fraction of mRNA between 56.3% and 93.5% (data not shown). However, after quality control, the disagreement between the transcriptomes at time points *t2.5*, *t10* and *t20* was higher than 30%. Consequently, these samples were considered outliers and were excluded from further analysis (Conesa *et al*., 2016). Therefore, T_1/2_ was calculated using only the time points *t0*, *t5*, *t40* and *t60*. The transcript abundance at time point *t0* (without rifampicin addition) was on average 6.1 ± 8.0 times lower than at time point *t5*. A similar pattern was observed in the transcript abundances of isolates where *t0* samples (without rifampicin) had on average 9.3 ± 7.4 times lower mRNA abundance than *t2.5* samples (Supporting Information Fig. S2). Lower abundance of transcripts prior to the addition of rifampicin has been reported for other bacterial cultures (Selinger *et al*., 2003; Steglich *et al*., 2010). These lags in transcription inhibition could result from the different rate of entry of rifampicin into the cells and/or from the insensitivity of RNA polymerase to rifampicin (Laguerre *et al*., 2018).

**Figure 2:**
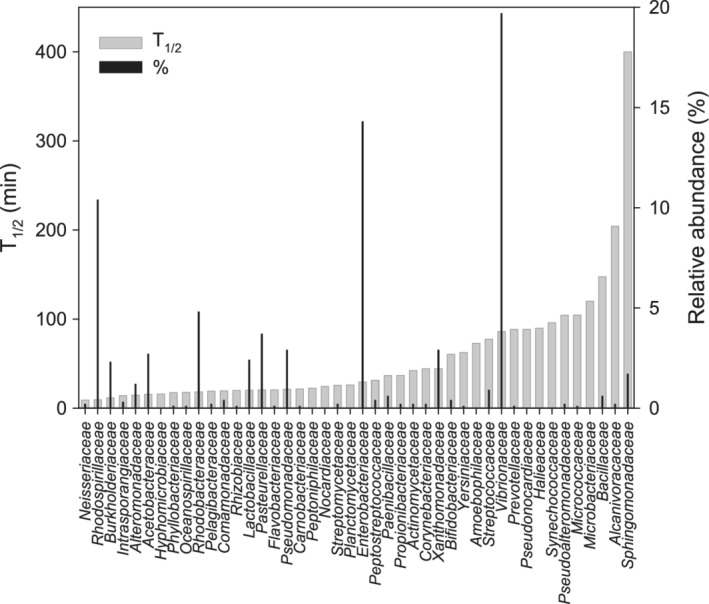
Half‐life time (T_1/2_) and relative abundance of rRNA (%) assigned to 44 bacterial families in the metatranscriptome from the coastal Adriatic Sea.

*Vibrionaceae* exhibited the highest relative transcript abundance (19.7 ± 3%) followed by *Enterobacteriaceae* (14.3 ± 2.4%) and *Rhodospirillaceae* (10.4 ± 0.9%) (Fig. 2), but no significant correlations between the relative abundance and T_1/2_ were found. The families *Vibrionaceae* and *Rhodospirillaceae* are among the most abundant bacterial families in the coastal northern Adriatic Sea (Steiner *et al*., unpublished), and frequently are the most active taxa in marine environmental communities (McCarren *et al*., 2010; Bergauer *et al*., 2018; Vorobev *et al*., 2018). The high transcript abundance of *Enterobacteriaceae* and other bacteria detected in this study might be due to the close vicinity of the sampling location to a fish cannery, releasing high amounts of untreated wastewater (Paliaga *et al*., 2017). In addition, the original bacterial community composition might have shifted during the five‐day incubation prior to initiating the experiment (Massana *et al*., 2001).

mRNA did not decay in 16 bacterial families in this study. The very slow or lack of decay might indicate resistance to the antibiotic rifampicin, as reported for clinical isolates and specific laboratory strains showing mutations in *rpo*B codons (Goldstein, 2014). *Mycobacterium tuberculosis* and *Staphylococcus aureus* of the families *Mycobacteriaceae* and *Staphylococcaceae*, respectively, have been reported to be resistant to rifampicin (Goldstein, 2014), in agreement with the lack of decay of mRNA of these two families (Supporting Information Table S2). Moreover, we could not determine mRNA decay rates for members of the orders *Burkholderiales*, *Pseudomonadales*, *Enterobacteriales*, *Actinomycetales*, *Rhizobiales* and *Shpingomonadales* (Supporting Information Table S2), as reported for soil isolates from these orders not only being resistant but can grow on several antibiotics as the sole carbon source (Dantas *et al*., 2008).

Exponential mRNA decay was determined in 44 families, with T_1/2_ ranging from 9 min to 400 with a median of 28 min (Fig. 2). The shortest T_1/2_ times, based on the whole transcriptome of a bacterial family, were longer than in the experiments with the bacterial strains, where decay rates of single gene transcripts of individual isolates were assessed. Most studies on RNA T_1/2_ are based on model organisms, such as *Escherichia coli* or *Bacillus subtilis*. The T_1/2_ of 329 known and predicted operons of *E. coli* vary strongly from less than 2 min to more than 20 min and rifampicin insensitivity has been reported (Selinger *et al*., 2003). Although about 80% of mRNAs of *Bacillus subtilis* have a T_1/2_ of less than 7 min, also extremely stable mRNAs have been detected in the same organism (Hambraeus *et al*., 2003). The transcriptome of *Enterobacteriaceae* (which includes *E. coli*) has a T_1/2_ of 29.5 min, while that of the family *Bacillaceae* (which includes *B. subtilis*) has a relatively long T_1/2_ of 147.5 min, indicating a faster mRNA turnover for *Enterobacteriaceae* in coastal Adriatic Sea waters.

**Figure 3:**
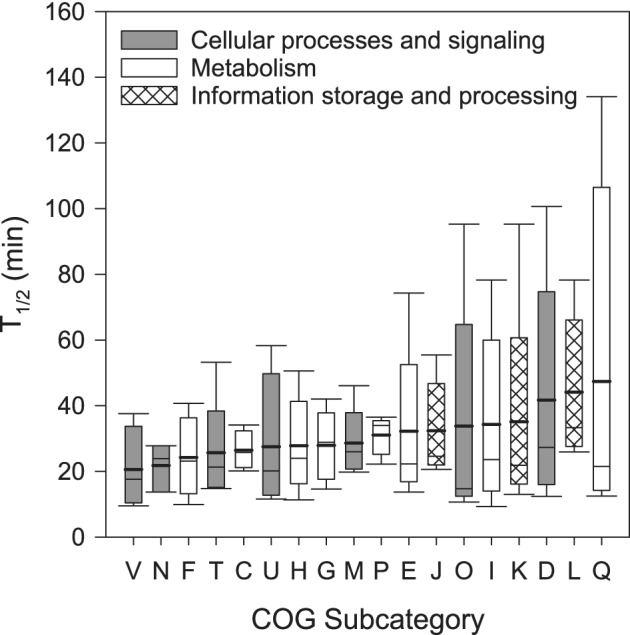
Half‐life time (T_1/2_) of COG categories and subcategories. The median is indicated by the thin horizontal line, the mean by the thick horizontal line. Whiskers indicate the 10th and 90th percentiles, and the edge of the box represent 25th, 75th percentiles. COG category ‘cellular processes and signalling’ includes the subcategories: **[V]** defence mechanisms, **[N]** cell motility, **[T]** signal transduction mechanisms, **[U]** intracellular trafficking, secretion, and vesicular transport, **[M]** cell wall/membrane/envelope biogenesis, **[O]** post‐translational modification, protein turnover, and chaperones and **[D]** cell cycle control, cell division, chromosome partitioning. COG category ‘metabolism’ includes the subcategories: **[F]** nucleotide transport and metabolism, **[C]** energy production and conversion, **[H]** coenzyme transport and metabolism, **[G]** carbohydrate transport and metabolism, **[P]** inorganic ion transport and metabolism, **[E]** amino acid transport and metabolism, **[I]** lipid transport and metabolism and **[Q]** secondary metabolites biosynthesis, transport and catabolism. COG category ‘information storage and processing’ includes the subcategories: **[J]** translation, ribosomal structure and biogenesis, **[K]** transcription and **[L]** replication, recombination and repair.

To address the variability of the T_1/2_ linked to different functional groups of mRNAs, the T_1/2_ was calculated for COG subcategories at the class taxa level. T_1/2_ of COG subcategories ranged from 9 to 134 min, with a median of 24 min (Fig. 3) and 28% of all T_1/2_ were shorter than 20 min. This substantially longer T_1/2_ as compared to previous reports on decay of single gene transcripts and single organisms (Hambraeus *et al*., 2003; Selinger *et al*., 2003; Hundt *et al*., 2007; Steglich *et al*., 2010; Kristoffersen *et al*., 2012) might be related to the broad grouping of bacteria into classes. However, mRNA assignments to lower taxonomic levels were generally too low to calculate T_1/2_ of COG subcategories, in agreement with the low levels of transcript abundance (< 1 per cell) for most genes recently reported in abundant marine bacteria (Cottrell and Kirchman, 2016).

**Figure 4:**
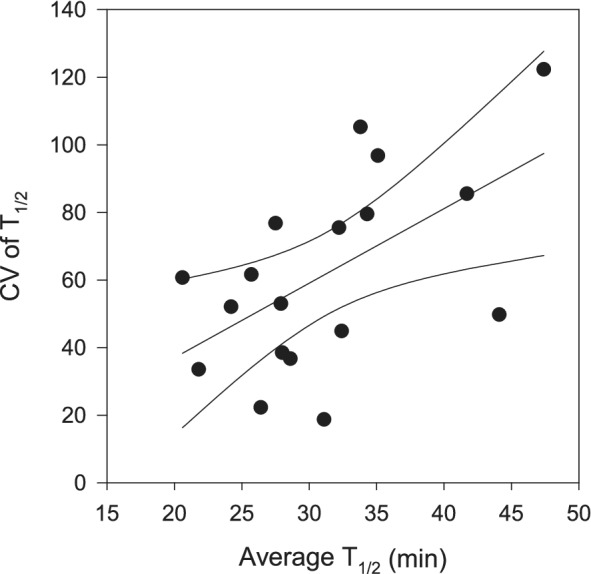
Linear regression with 95% confidence intervals between the coefficient of variance (CV) of the half‐life time (T_1/2_) and the average half‐life time of COG subcategories based on the five most abundant bacterial classes (*Gammaproteobacteria*, *Alphaproteobacteria*, *Bacilli*, *Betaproteobacteria* and *Actinobacteria)*. Based on 18 COG categories, the correlation coefficient was *r* = 0.56 and *p‐*value = 0.01.

The average T_1/2_ and the coefficient of variance (CV) of the different COG subcategories were weakly positively correlated (*r* = 0.56, *p* = 0.01, *n* = 18), indicating that COG subcategories with shorter T_1/2_ had a less variable mRNA T_1/2_ in the five bacterial classes analysed than subcategories with longer T_1/2_ (Fig. 4). The significance of a conserved short T_1/2_ in specific functions derives from the necessity of rapidly responding to changes in the environment (Takayama and Kjelleberg, 2000). The transcripts from information storage and processing COG category generally had long T_1/2_, while cellular processes and signalling and metabolism had variable T_1/2_ (Fig. 3). The shortest T_1/2_ corresponded to the COG subcategories related to defence mechanisms and cell motility (Fig. 3). The four most abundant COGs within the defence mechanisms were ABC transporters of the ABC‐type multidrug transport system involved in the transport of nutrients or toxins through the cell membranes (Davidson *et al*., 2008). The most frequently expressed gene within the cell motility COG subcategory was the protein flagellin, a component of the bacterial flagellum (Vonderviszt and Namba, 2013). These results suggest that shorter T_1/2_ of mRNA and thus, rapid turnover time prevail for functions related to the response of the cells to environmental conditions, such as patches of nutrients stimulating chemotactic responses or presence of toxins. The widespread pattern in the five bacterial classes analysed here further indicate the significance of these functions for the survival and fitness of most, if not all, bacteria in the environment.

### *Conclusions*

Overall, the mRNA T_1/2_ of 11 genes of the three marine bacterial isolates grown at different temperatures was commonly shorter than 10 min, supporting the need of fast processing of samples when studying transcription in natural communities. Sample collection of surface waters (100 to 200 m) with traditional oceanographic methods (e.g. Niskin bottles) can take as long as 30 min, allowing for substantial, possibly stress‐induced changes in the metatranscriptome (Edgcomb *et al*., 2016). A variety of recently developed *in situ* samplers, implemented with optional RNA fixation mechanisms, permit a more reliable sampling of environmental RNA (Feike *et al*., 2012; McQuillan and Robidart, 2017). Nevertheless, activity profiling of microorganisms based on mRNA remains questionable due to the poor correlations of mRNA with protein levels (Greenbaum *et al*., 2003; Wang *et al*., 2019). Recently, it has been shown that eukaryal and archaeal mRNA have a longer T_1/2_ as compared to bacterial mRNA (Clouet‐d'Orval *et al*., 2018). Hence, care has to be taken when drawing conclusions based on metatranscriptomes, likely including organisms from the three domains of life. Furthermore, we demonstrate here that the T_1/2_ of mRNA varied widely within a complex environmental bacterial community, potentially biasing the interpretation of activity profiles.

Different factors affected the half‐life time of RNA in the marine bacterial isolates and the bacterial community studied here. There were clear differences in T_1/2_ of RNA with distinct functions (mRNA *vs*. ncRNA) likely due to differences in physical protection (Deutscher, 2003, 2006). Moreover, short mRNA T_1/2_ prevails in genes related to the response to environmental stressors. However, the relationship of T_1/2_ with temperature and growth rates varied among the different isolates, suggesting that these two factors interact affecting RNA degradation. Our findings emphasize the need to consider the short T_1/2_ and its high variability when assessing metatranscriptomes especially in environmental samples and highlight the requirement to minimize processing and handling time.

## Experimental procedures

### *Sample collection, bacterial isolation and sequencing*

The bacteria *Alcanivorax jadensis*, *Croceibacter atlanticus* and *Colwellia polaris* were isolated from the mesopelagic (250 m) and bathypelagic (3200 m) North Atlantic (67.35°N 4.94°W) and from the surface waters (~2 m) of the Adriatic Sea off the coast of Piran (Slovenia), respectively. Seawater from the different locations was amended with media (15 g/l Select Agar and 25 g/l LB Broth), autoclaved and distributed in petri dishes. Fifty microliters of the respective seawater was spread on the plates and incubated in the dark at 20 °C. Three of the growing bacterial colonies were picked with a sterile toothpick and preserved after flash‐freezing in liquid N_2_ for subsequent experiments. The 16S rRNA gene from the different isolates was PCR amplified using the primers 27F (5’ AGAGTTTGATCCTGGCTCAG 3′) and 1492R (5’ GGTTACCTTGTTACGACTT 3′) and the following thermocycling conditions: 94 °C for 4 min, 30 cycles of denaturation at 94 °C for 1 min, annealing at 55 °C for 1 min and extension at 72 °C for 1 min, followed by a final extension at 72 °C for 30 min and hold at 4 °C. PCR products were checked on a 2% agarose gel, stained with SYBR Gold (Invitrogen) and purified using a PCR extract mini kit (5‐PRIME). The DNA concentration from the PCR products was quantified on a Nanodrop 2000 spectrophotometer (Thermo Scientific) prior to Sanger sequencing (Applied Biosystems 3130 × l genetic analyser). Sequences were analysed using FinchTV and aligned with CodonCode Aligner. Taxonomic identification was conducted with BLAST against the NCBI nucleotide database.

### *Growth characteristics of the different isolates*

The three bacterial isolates were inoculated in duplicate 50 ml LB liquid media and incubated at 4, 10, 15, 20, 25 and 30 °C. Growth rates and growth stages (lag, log, stationary and death phase) of the different isolates were determined for each temperature. Subsamples for bacterial abundance (1 ml) were taken at approximately 12, 8 and 4 h intervals, depending on the growth curve for the specific culture and temperature. Subsamples for bacterial abundance were fixed with glutaraldehyde (0.5% final concentration) and kept at 4 °C in the dark for 10 min. Subsequently, the samples were flash‐frozen in liquid nitrogen and stored at −80 °C. Prior to cell enumeration, samples were diluted 1:10 with TRIS‐EDTA‐buffer and stained with SYBRGreen I (Invitrogen, 1× final concentration) in the dark for 10 min. Polystyrene 1 μm beads (FluoSpheres®, Fisher scientific, ~ 10^5^ beads mL^−1^ final concentration) were added as an internal standard. Afterwards, samples were vortexed and run in an Accuri C6 flow cytometer (BD Biosciences). Bacterial cells were distinguished based on their green *versus* side scatter signals (Brussaard, 2004).

The generation time (*G*) was calculated as follows: G = *t* / ((log(N)‐log(N0))/log(2)) where *t* is the time from the beginning of the exponential phase to the beginning of the stationary phase; *N0* is the cell abundance at the beginning of the exponential phase; and *N* is the cell abundance at the beginning of the stationary phase.

### *RNA degradation experiment using bacterial isolates*

The RNA degradation experiment started at the beginning of the stationary phase for each of the bacterial isolates growing at 4, 15 and 25 °C. The three bacterial isolates were inoculated in duplicate 50 ml liquid media. Rifampicin was used to inhibit bacterial DNA‐dependent RNA polymerase (Calvori *et al*., 1965) in order to determine RNA half‐life time (Hambraeus *et al*., 2003; Selinger *et al*., 2003; Steglich *et al*., 2010). An initial *t0* subsample (1 ml) was taken in duplicate prior to the addition of rifampicin and used as a control. Then, 150 μg/ml rifampicin (final concentration) was added to the 50 ml liquid duplicate cultures. One millilitre of subsamples were collected after 2.5, 5, 10, 20, 40 and 60 min of incubation (subsequently referred to as *t2.5*, *t5*, *t10*, *t20*, *t40* and *t60*) following the rifampicin addition. All subsamples were immediately fixed with 1 ml RNA later (Invitrogen) and flash‐frozen in liquid nitrogen.

### *RNA degradation experiment using a complex coastal bacterial community*

Seawater was collected with an HCl cleaned 10 l carboy from the coastal northern Adriatic Sea at Valdibora Bay (Rovinj, Croatia) on 07 Nov 2016. The collected water was pre‐filtered through a sterile 0.8 μm polycarbonate filter (Millipore) with HCl cleaned tubes and filtration devices. The 0.8‐μm filtered seawater was stored at 20.5 °C in the dark for five days to allow acclimatization of the prokaryotic community. The first sample was taken prior to the addition of rifampicin (initial RNA *t0*) and used as a control. After adding rifampicin to 1.2 l of seawater (150 μg/ml final concentration), samples were taken at 0, 2.5, 5, 10, 20, 40 and 60 min (subsequently referred to as time point *t0*, *t2.5*, *t5*, *t10*, *t20*, *t40* and *t60*). At each sampling, 150 ml were collected and fixed with 150 ml RNA storage solution (200 mM sucrose, 5 mM EDTA, 10 mM sodium acetate pH 5.2) (Steglich *et al*., 2010) and subsequently filtered onto 0.2 μm Millipore GTTP filters. Immediately after filtration, the filters were flash‐frozen in liquid nitrogen and stored at −80 °C.

### *RNA extraction and cDNA preparation*

The pipets and the surface of the lab bench used during the extractions were cleaned with 70% ethanol and RNase Killer (5Prime) prior to use. All the material used was RNase/DNase free and all the stock buffer solutions and water were pretreated with diethyl pyrocarbonate (DEPC). RNA was extracted using a hot phenol/chloroform extraction modified from Kramer *et al*. (1996). Detailed information is available in the Supplementary Material (Appendix S1). No negative controls for RNA extraction and sequencing were included in this study.

### *Primer design, PCR and qPCR*

Taxa‐specific primers were designed for genes encoding proteins that belonged to distinct categories or subcategories to cover a diversity of protein functions. In order to choose genes that are present in most bacterial cells, genes predicted to be a part of the core gene set of bacterial cells were selected (Gil *et al*., 2004). The selected genes were ATP synthase B chain (*atp*F), chaperone Hsp70 (*dna*K), cytoskeletal cell division protein (*fts*Z), ATP‐dependent protease (*hfl*B), aminopeptidase A/I (*pep*A), pyruvate kinase (*pyk*A), clp protease subunit 2 (*clp*P), phosphatidylserine synthase (*pss*A), RNA polymerase ß subunit (*rpo*B) and thymidylate synthase (*thy*A). Primer sets were designed for all the genes and isolates, however, only those that successfully amplified the specific gene are shown in Supporting Information Table S3. The isolates were identified solely based on their 16S rRNA sequences. Subsequently, we used available data on full‐genome sequenced cultures that corresponded to our isolates according to the identity of the 16SrRNA and on other close relatives. However, some variation in the genome of the isolates might occur compared to the available data. Potentially, variation in some positions of the primer sets designed might have hindered efficient amplification of some of the genes and isolates. The taxa‐specific primers were designed in Geneious 6.1.8 (Biomatters, Ltd) using the sequences from close relatives to the isolates (Supporting Information Table S4). Specificity was confirmed by gel electrophoresis using the extracted DNA from the isolates. 16S rRNA (Suzuki *et al*., 2000) and *rec*A (Holmes *et al*., 2004) genes were amplified with available non‐taxa specific primer sets (Supporting Information Table S3). Yet, the *rec*A primer set only successfully amplified the *Colwellia* isolate. The corresponding amplified fragments were kept as short as possible to minimize PCR efficiency differences (Debode *et al*., 2017). Due to the varying location of conserved sites in the different genes, however, fragment size varied between 123 and 532 bp (Supporting Information Table S3). Regardless of the different fragment size, PCR efficiency varied between 68.7% and 98.9% (Supporting Information Table S3).

Annealing temperatures for all specific primers were determined by gradient PCR, and the amplified products were checked by electrophoresis on a 2% agarose gel. The annealing temperatures with best results were used in qPCR (Supporting Information Table S3). Standard dilutions from 10^7^ to 1 gene for quantification were prepared from purified PCR products of the isolated bacteria as previously described (Sintes *et al*., 2013). The standard dilution was loaded to each qPCR plate (Bio‐Rad) together with the cDNA samples, RNA samples, a positive and a negative control. All samples were loaded in triplicate and the plates were closed with optical tape (Bio‐Rad) and run on a Light Cycler 480 (Roche). Successful amplification in the RNA samples indicates the presence of undigested DNA. On average, 5% ±11% SD of cDNA gene abundance corresponded to undigested DNA. The gene abundance in the RNA sample was subtracted from cDNA gene abundance. The reaction mixture for each sample contained 1× Mastermix (LightCycler 480 SYBRGreen I Master, Roche), 0.5 μM of the forward and reverse specific primers, 1 μl of sample and ultrapure sterile water (Roche) up to 10 μl. Thermocycling for all genes was initiated by a denaturation step at 95 °C for 10 min, followed by 50 cycles consisting of a denaturation step at 95 °C for 5 s; annealing temperatures and times for each gene as listed in Supporting Information Table S3; extension at 72 °C for 15 s and a plate read at 74 °C for 3 s.

### *Metatranscript library preparation*

The transcribed cDNA obtained from the natural bacterial community was fragmented (~500 bp) with a Covaris focused‐ultrasonicator. The concentration of the obtained cDNA fragments was measured with a QUBIT spectrophotometer (ThermoFisher) following the manufacturer's protocol. The library preparation was performed with a KAPA Hyper kit (KAPA Biosystems). Briefly, 10 ng of cDNA were end repaired and A‐tailed. Subsequently, the genetic material was ligated and purified using a bead‐based cleanup method. The obtained libraries were PCR amplified with following conditions: 98 °C for 45 s, 12 cycles of denaturation at 98 °C for 15 s, annealing at 60 °C for 30 s and extension at 72 °C for 30 s, followed by a final extension at 72 °C for 1 min and hold at 4 °C. The PCR products were further purified using a bead‐based cleanup method. The libraries concentrations were measured by qPCR (KAPA hyper kit) following the manufacturer's recommendations, and 2.5 nM cDNA of each sample was sequenced using an Illumina MiSeq high throughput sequencing (2 × 250 paired‐end platform) at JAMSTEC, Japan. The sequence data generated are publically available in the DDBJ sequence read archive (DRA) under the accession number DRA008144 for sample *t*0, DRA008145 for sample *t*5, DRA008146 for sample *t*40 and DRA008147 for sample *t*60.

### *Bioinformatics and statistical analyses*

The transcriptomes were analysed using a read‐based approach. The program SortmeRNA (Kopylova *et al*., 2012) was used to sort mRNA reads from rRNA reads with all available databases (RFAM (December 22, 2015) and SILVA (July 23, 2015): rfam‐5.8s‐database‐id98.fasta, rfam‐5s‐database‐id98.fasta, silva‐arc‐16s‐id95.fasta, silva‐arc‐23s‐id98.fasta, silva‐bac‐16s‐id90.fasta, silva‐bac‐23s‐id98.fasta, silva‐euk‐18s‐id95.fasta and silva‐euk‐28s‐id98.fasta). Adapters were identified and removed with AdapterRemoval version 2.1.7 and the following settings: ‐‐identify‐adapters, ‐‐file1, ‐‐file2) (Schubert *et al*., 2016). Paired‐end reads were merged with PEAR (v0.9.10) (Zhang *et al*., 2013) and diamond (Buchfink *et al*., 2014) was performed using the nr database (April 2018) for transcriptome annotation with the following settings: ‐b 12.0, ‐e 0.00001, ‐k 1. The diamond blast results were analysed with MEGAN6 v6.13 (Huson *et al*., 2007). Taxonomic affiliation was assigned using the database prot_acc2tax‐Oct2017X1.abin and the ‘Naive LCA’ algorithm, with default parameters. Functional assignment was assessed with the database acc2eggnog‐Oct2016X.abin. The absolute comparison mode in EGGNOG Viewer (Powell *et al*., 2014) of MEGAN6 was used to extract functional gene counts with the ‘Best‐Hit’ algorithm for binning. Further analysis was performed only in transcriptomes keeping more than 50% of the original mRNA read abundance after quality control.

The transcriptomes were analysed using two approaches. The first approach consisted in determining the T_1/2_ of bacteria at the ‘family’ taxonomic level. Functional genes (mRNA) were successfully assigned to 112 families (127,472 assignments), while phylogenetic genes (16S rRNA gene) were successfully assigned to 182 families (186,993 assignments) with MEGAN6. Functional and phylogenetic genes could be jointly assigned to 100 families. Forty families were excluded from further analysis due to ineffective detection in all time points, and 16 families due to non‐exponential decay of their mRNA. Consequently, 44 families were used for T_1/2_ calculations.

The second approach consisted in determining the T_1/2_ of functional categories from bacterial taxonomic classes. We used the EggNOG database (Huerta‐Cepas *et al*., 2015) and COG categories (Tatusov *et al*., 1997; Galperin *et al*., 2014) to classify the mRNA. Five bacterial classes (*Gammaproteobacteria*, *Alphaproteobacteria*, *Bacilli*, *Betaproteobacteria* and *Actinobacteria*) comprised more than 1000 rRNA assignments and more than 200 mRNA assignments on average. These were subsequently used to calculate the T_1/2_ of COG subcategories.

The relative abundance of transcripts was assessed as the ratio of mRNA to rRNA at each time point for both approaches. Therefore, the number of mRNA assignments was divided by the number of rRNA assignments to a bacterial family or COG category. The relative abundance of bacterial families was calculated by dividing the number of rRNA assignments to each bacterial family by the total rRNA in each sample. The mean and standard deviation were calculated for each family for all merged samples. Tests of significance were calculated with Mann–Whitney pairwise test and one‐sample *t*‐test using the program Past3 v3.20 (Hammer *et al*., 2001).

### *RNA half‐life time (T_1/2_)*

T_1/2_ was calculated with the ‘relative two phase decay model’ according to Steglich *et al*. (2010). Briefly, an exponential decay was fitted to the RNA expression values *versus* time after rifampicin addition. The time point with maximal expression (N_0_) was selected as the initial time point of the decay (*t*_0_). The expression value (N) at the last time point (*t*) of the exponential decay was chosen that the best fit was achieved: T_1/2_ = (*t* – *t*_0_) / (log_2_ (N_0_) – log_2_ (N)). This method was chosen to target the previously described two phases of RNA decay (delay and decay phase) (Steglich *et al*., 2010).

## Competing interests

The authors declare that they have no competing interests.

## Funding

This study was funded by the Wittgenstein prize (Austrian Science Fund, FWF, Z194‐B1) to G.J.H. and the projects ARTEMIS (P28781‐B21) and DK microbial nitrogen cycling (W1257‐B20) to G.J.H., and by the project of the FWF P27696‐B22 to E.S. Part of the laboratory work was supported by the JSPS KAKENHI Grant Number 16F16085. D.D.C. was supported by an Overseas Researcher Grant under Postdoctoral Fellowship of Japan Society for Promotion of Science (P16085).

## Authors' contributions

E.S. conceived and together with D.D.C. and P.A.S carried out the experiments. P.A.S. analysed the data and wrote the paper. P.A.S and C.M. extracted RNA. E.S. designed qPCR primers. P.A.S. carried out qPCR. D.D.C. prepared the metatranscript library and T.Y. coordinated and supervised the sequencing. J.G. and T.R. provided bioinformatics support. G.J.H. and E.S. provided project oversight. All authors read and approved the final manuscript.

## Supporting information


**Appendix S1:** Supporting information

**Figure S1**. Development of the cell abundance of the bacterial strains *Alc*: *Alcanivorax jadensis*, *Col: Colwellia polaris* and Cro: *Croceibacter atlanticus* grown in duplicates at 4, 15 and 25°C.

**Figure S2**. Transcript abundance of different genes over time of the bacterial isolates *Alcanivorax jadensis* (n = 9), *Colwellia polaris* (n = 15) and *Croceibacter atlanticus* (n = 21) at 4, 15 and 25°C. Average ± standard deviation (SD) of triplicate measurements is shown.

**Figure S3**. Generation time of the bacterial isolates *Alcanivorax jadensis*, *Colwellia polaris* and *Croceibacter atlanticus* grown at 4, 10, 15, 20, 25 and 30°C.

**Table S1**. Correlation coefficient (r) and p value between half‐life time (T_1/2_), temperature (°C) and generation time (g) of each gene and bacterial isolate. *P* values < 0.05 are in bold.
**Table S2**. Bacterial families with non‐detectable mRNA decay. The taxonomic levels order and phylum and relative abundance (% of total rRNA) at time point T0 are indicated.
**Table S3**. Primer sets (F: forward, R: reverse), and annealing conditions used for q‐PCR of different functional genes in the different isolates. Estimated average amplification efficiency (%) and length of the fragment are indicated. Only primer sets successfully amplifying the respective transcript are shown for each isolate.
**Table S4**. Gene bank accession numbers for the sequences used to design taxon specific primer sets.

## Data Availability

The datasets generated and/or analysed during the current study are available in the DDBJ Sequence Read Archive (DRA): http://ddbj.nig.ac.jp/DRASearch/
