# Supplementary material for: Highly variable mRNA half‐life time within marine bacterial taxa and functional genes
Source: Environ Microbiol. 2019 Jul 25;21(10):3873–84. doi: 10.1111/1462-2920.14737 (PMC7379614; doi:10.1111/1462-2920.14737)
Supplement: Supplementary file 1 — Appendix S1: Supporting information [file EMI-21-3873-s002.docx]

**Supplementary materials**

*RNA extraction and cDNA preparation*

Lysis of bacterial cells was initiated by thawing the RNA Later preserved subsamples and concentrating them to 100 µL by centrifugation at 3220x *g* and 4°C with 10 KDa Amicon Ultra 2 mL Centrifugal filters (Ultracel 10K, Millipore). Samples of the RNA degradation experiment with a complex coastal bacterial community were collected on filters and flash-frozen. For RNA extraction, the filters were thawed and cut into small pieces and subsequently incubated with the lysis buffer. Lysis buffer (0.1 mL Tris 100 mM pH 8, 0.1 mL EDTA 250 mM pH8, 0.02 mL NaCl 5 M, made up to 1 mL with water) and lysozyme solution (8.75 U/mL final concentration) (Lysozyme Ready-Lyse, Epicenter) were added to the concentrated isolate or the filter pieces. Samples were incubated at 37°C for 45 min. Thereafter, SDS (final conc.: 1 %) and proteinase K (final conc.: ~8 U/mL) (Proteinase K from Tritirachium album, Sigma-Aldrich) were added and incubated at 55°C for 1h. Thereafter, combusted zirconium beads ~200 µL (0.1 mm diameter Zirconia/Silica, BioSpec Products) were added. The samples were subsequently vortexed for 10 min at maximum speed and incubated at 70°C for 30 min. The lysate (supernatant) was recovered after centrifugation at 4500x *g* for 5 min and pipetted into clean 2 mL Eppendorf tubes. After the lysis, 90 µL of 1 M NaOAc (pH 5.2) per tube (final conc. 100 mM) and an equal volume (~1 mL) of water saturated phenol (pH 4.3, Sigma-Aldrich) were added, mixed with the lysate and incubated at 64°C for 6 min. Samples were chilled on ice and centrifuged at 21,000x *g* at 4°C for 15 min. The aqueous layer was transferred to a Phase Lock Gel (Phase Lock Gel Heavy 2 mL, 5Prime) with an equal volume of chloroform (~0.9 mL) (Sigma-Aldrich) and centrifuged at 21,000x *g* at 4°C for 10 min. The aqueous layer was split into two 1.5 mL eppendorf tubes and ethanol precipitation was initiated by adding 1/10 volume of 3 M NaOAc (pH 5.2), 1 mM EDTA and 2 volumes cold ethanol (for molecular biology, Merck) followed by incubation at -80°C overnight. Subsequently, samples were thawed and centrifuged at 21,000x *g* at 4°C for 25 min. The pellets were washed with 1 mL ice cold 80% ethanol and centrifuged at 21,000x *g* at 4°C for 20 min. Afterwards, the pellets were air-dried at 45°C for 3-4 h and re-suspended in 100 µL DEPC-water at 4ºC overnight (10-18h). Thereafter, all the RNA extracts from the same sample were combined into one microfuge tube and stored at -80°C until further analysis. Residual DNA from the nucleic acid extract was digested and the RNA was purified with RNeasy MiniElute Cleanup Kit (RNase free DNase set, Qiagen) following the manufacturer’s protocol. Elution was carried out twice with 14 µL RNAse/DNAse free water (final volume of ~28 µL of eluate). RNA concentration and quality were determined spectrophotometrically with a Nanodrop 2000 (Thermo Scientific) and subsequently by electrophoresis with an Experion (Bio-Rad) using the RNA HighSens Kit (Bio-Rad). RNA quality is indicated by the 23S rRNA to 16S rRNA ratio (Sambrook et al., 1989). However, it has been shown that this method might be misleading (Bhagwat et al., 2013), hence we mainly checked the quality by visual inspection of the electropherograms. Purified RNA was transcribed into cDNA using Super Script III First-Strand Synthesis System (Invitrogen) with random hexamers. The resulting cDNA was diluted 1:10 prior to qPCR analysis.

**References**

Bhagwat, A.A., Ying, Z.I., Karns, J., and Smith, A. (2013) Determining RNA quality for NextGen sequencing: some exceptions to the gold standard rule of 23S to 16S rRNA ratio. *Microbiology Discovery* **1**: 10.

Sambrook, J., Fritsch, E.F., and Maniatis, T. (1989) *Molecular cloning: a laboratory manual*. Cold Spring Harbor, NY: Cold Spring Harbor Laboratory Press.
